# Effectiveness of a Game-Based Mobile App for Educating Intensive Critical Care Specialist Nurses in Extracorporeal Membrane Oxygenation Pipeline Preflushing: Quasi-Experimental Trial

**DOI:** 10.2196/43181

**Published:** 2023-12-07

**Authors:** Zihan Wang, Ruting Gu, Jingyuan Wang, Yubiao Gai, Hui Lin, Yan Zhang, Qianqian Li, Tong Sun, Lili Wei

**Affiliations:** 1 School of Nursing, Qingdao University, Qingdao, China; 2 Nursing Department, The Affiliated Hospital of Qingdao University, Qingdao, China; 3 Population Monitoring and Family Development Department, Qingdao Licang Municipal Health Commission, Qingdao, China

**Keywords:** extracorporeal membrane oxygenation, mobile app, simulation training, specialist nurses, learning curve, ECMO, intensive care

## Abstract

**Background:**

In the context of training specialist nurses and nursing education, a game-based mobile app was used as a simulation to teach intensive critical care specialist nurses the knowledge and skills of extracorporeal membrane oxygenation (ECMO) pipeline preflushing.

**Objective:**

This study aimed to evaluate the impact of a game-based mobile app on improving ECMO pipeline preflushing skills in intensive critical care specialist nurses.

**Methods:**

A total of 86 intensive critical care specialist nurses who were learning ECMO for the first time were included in this study. The nurses were divided into 2 groups: a control group (n=43) and an experimental group (n=43). Participants in the experimental group used a game-based mobile app for simulation exercises; the control group received no additional intervention. All participants took a theoretical test and a skill operation test at the beginning of the study and 1 week later. The differences in scores between the 2 groups were compared, and the learning curve of the experimental group was observed.

**Results:**

The final theoretical test scores (88.44 and 85.02) and skill operation test scores (89.42 and 86.33) of the experimental group and control group, respectively, were significantly higher than those of the initial tests (theoretical test scores: 75.88 and 74.42; skill operation test scores: 75.44 and 75.93; all *P*<.001). The scores of the final theoretical test (88.44) and the final skill operation test (89.42) in the experimental group were higher than the scores of the control group (85.02; *P*<.001 and 86.33; *P*<.001, respectively). Learning curve analysis showed that the experimental group needed an average of 17 operations to master the skill.

**Conclusions:**

This study suggests that a game-based mobile app may be more effective for intensive critical care specialist nurses in ECMO pipeline preflushing education than traditional Chinese lecture-practice education.

## Introduction

Extracorporeal membrane oxygenation (ECMO) is a treatment that uses an improved extracorporeal circulation system to provide temporary life support for patients with potentially reversible heart or respiratory failure [1]. ECMO achieves the purpose of lung or heart recovery from disease by providing a mechanism for gas exchange and heart support. With the continuous development of ECMO-related technologies, it has become a recognized treatment method for neonates, children, and adults who do not respond to conventional medications for respiratory failure and heart failure. According to statistics, among 1099 patients with COVID-19 in China diagnosed between December 2019 and January 29, 2020, a total of 55 patients entered the intensive care unit for treatment, 67 patients underwent invasive mechanical ventilation, and 5 patients received ECMO treatment [2]. During the new coronary pneumonia epidemic, the application of ECMO played an important role during the treatment of patients who are critically ill. Despite major advancements in technology, ECMO is still associated with high morbidity and mortality [3,4], with nosocomial infections being the leading causes, including ventilator-associated pneumonia (57.6%), tracheobronchitis (9.1%), bloodstream infections (9.1%), skin and soft tissue infections (9.1%), and cytomegalovirus reactivation (9.1%) [5]. Although the occurrence of these complications is not necessarily directly related to improper pipeline preflushing, if medical staff do not follow the correct sequence of steps and do not observe the principle of sterility, it may lead to an increased risk of complications. Therefore, it is necessary to train intensive critical care specialist nurses on ECMO.

In recent years, to compensate for the shortcomings of traditional education models, such as classroom teaching and group exercises, and to improve teaching quality, education in the field of medicine and health has continuously sought more innovative models and methods. More and more educators are applying game teaching to medical field teaching. For example, Tan et al [6] applied games to blood transfusion teaching and found that they had a positive effect in improving nursing students’ knowledge and confidence in blood transfusion practice. Researchers used video games to simulate nursing and medical students’ first visit to the operating room in hopes of improving perception and performance and reducing stress [7]. In addition, a serious game was used to supplement the teaching of cardiopulmonary resuscitation (CPR) [8]. This game could simulate emergencies in a 3D virtual environment. Participants must apply CPR to rescue an injured patient. The use of this game improved students’ CPR knowledge and skills [8]. At present, there are few innovative education studies on ECMO and its pipeline preflushing; they mainly consist of the combination of education using high-fidelity simulation and augmented reality (AR) technology. Researchers have carried out high-fidelity simulation education and training courses for intensive critical care specialist nurses, improving their personal skills and the skill level of the whole team [1,9]. There was also great potential for improving the effect of ECMO intubation training based on AR instructions [10]. However, due to the high cost of training using the abovementioned teaching methods or techniques, and limited by the training venue and practice time, students usually do not have enough time to practice and cannot check the learning practice results in a timely manner.

The traditional ECMO training method mainly consists of students or nurses passively accepting theoretical explanations and technical operation demonstrations. The training method is simple and lacks innovation, and the training process does not evoke enthusiasm and participation from the trainees. In addition, the traditional ECMO training method, including high-fidelity simulation and AR technology education, limits the time and place for nurses to perform operational exercises [1,9]. To our knowledge, there have been no studies on the use of games in ECMO education or ECMO pipeline preflushing teaching. Therefore, this study integrated games into traditional clinical teaching methods and developed a game-based mobile app of ECMO pipeline preflushing, which enables intensive critical care specialist nurses to master the relevant technologies; at the same time, the app can become a tool for nurses to learn relevant knowledge and test learning results anytime and anywhere. We explored whether a game-based mobile app affected the practical knowledge and skill levels of intensive critical care specialist nurses. At the same time, combined with the learning curve, we analyzed the minimum number of operations intensive critical care specialist nurses needed to master this operation, to provide a reference for formulating more standardized training and guiding ECMO education in the future.

## Methods

### Participants

The study population included 86 nurses who were undergoing targeted training as intensive critical care specialist nurses at a university-affiliated hospital in China.

The inclusion criteria for this study consisted of the following:

- Having a mobile phone or computer
- Having internet access
- Having obtained the intensive critical care specialist nurse certificate but no previous experience in ECMO

The exclusion criteria for this study consisted of the following:

- Having participated in at least 1 theoretical lecture, demonstration, or group study on ECMO
- Have not undertaken the first or final theoretical and skill performance assessments of ECMO pipeline preflushing

Based on the experimental results, the researchers determined that the required number of nurses was at least 68 intensive critical care specialist nurses (34 in the experimental group and 34 in the control group). A total of 86 participants (43 in the experimental group and 43 in the control group) were included in the study due to an expected loss of 10% to 20% of the sample during follow-up.

We used convenience sampling methods to select intensive critical care specialist nurses from 2 districts (the eastern district and the western district) of a university-affiliated hospital in China as the research participants. Among them, those from the western district were used as the experimental group, and those from the eastern district were used as the control group. We registered the participants’ details and kept the group information confidential. Before completing the training and evaluation, the trainers and participants were not informed of the group allocation.

### Ethical Considerations

This study was approved by the Medical Ethics Committee of the Affiliated Hospital of Qingdao University, where the researchers are located. In this project, the rights and interests of the participants were fully protected, and the research protocol was approved in accordance with the requirements of the Medical Ethics Committee (approval number: QYFYWZLL26706). In this study, all participants provided informed consent, and participants reserved the right to withdraw at any time. During data collection and analysis, the data were anonymized, and only a number was used to match the results of each test. No other form of compensation was provided to the participants in this study.

### Procedures

The study was conducted from March 8, 2021, to March 18, 2021. To prevent interaction between the 2 groups, intensive critical care specialist nurses from 2 different districts of the same general hospital were selected for the study. The 2 districts are located on opposite sides of Jiaozhou Bay, 40 km apart. The same management team was responsible for the management and training of the 2 groups.

On March 8, 2021, all intensive critical care specialist nurses conducted a 1-day theoretical training in the Shinan district of this general hospital. The training content referred to the *ECMO Manual* (2nd edition; Chinese version) [11] and *ELSO Guidelines for Training and Continuing Education of ECMO Specialists* [12], including the principles and indications of ECMO, contraindications, equipment and consumables, pipeline preflushing, etc. Subsequently, all intensive critical care specialist nurses underwent 2 days of skill training and practice. The skill training corresponded to the content of theoretical training. After the 3 days of on-site intensive training, the first theoretical and skill tests were immediately carried out. The intensive critical care specialist nurses who participated in the tests were not known to the examiners; they knew only that all participants had received training on ECMO.

Next, we downloaded the game-based ECMO pipeline preflushing mobile app onto the mobile phones of the intensive critical care specialist nurses of the experimental group, explained that they could play the game anytime and anywhere in the next 7 days, and asked them to use and complete the program more than 30 times. Within 7 days after the first evaluation, the control group received no additional intervention measures. Seven days after the intervention measures were implemented, the 2 groups of participants were again assessed on theoretical knowledge and skills.

### Preparation of the Game-Based Mobile App

The game-based mobile app was designed and developed by a team of authors, including 6 nursing administrators with more than 10 years of clinical teaching experience, 10 clinical teachers, 1 software engineer, 1 technical support staff, 1 artist, and 1 graduate nurse. The software research and development team made reference to previous literature—based on the training of applied skills in nursing and combined with clinical teaching experience, gamification teaching theory, and the use of virtual clinical resources as the carrier—and simulated a clinical operation with high adaptability [13]. The researchers designed an “ECMO Pipe Preflushing Skill List” based on the *ECMO Manual* (2nd Edition; Chinese version) [11] and used it to create a game scene including pictures and animations. The researchers used the C++ programming language, which can be used to write a working system language or application programming languages [14], and the Cocos2D game engine to develop the game-based mobile phone app [15]. During software development, the team was responsible for overall coordination, supervision, management, and quality control, and they recorded, summarized, and organized the data for the entire process. This app was reviewed by professionals with a medical background. Since the software works with any Android operating system, the app can be played anywhere without an internet connection. Nurses start the game by registering with their phone number, but at first, they need to be connected to the internet to download the game.

According to the sequence of operations, the game was divided into 7 stages with a total of 61 steps:

- Stage 1: Preparing items and materials according to the detailed list (13 steps)
- Stage 2: Checking supplies (5 steps)
- Stage 3: Connecting and installing pipelines (12 steps)
- Stage 4: Using gravity to preflush the front membrane pipeline (4 steps)
- Stage 5: Using the machine to exhaust the gas after the membrane (17 steps)
- Stage 6: Stopping the pump, clamping the pipeline, and removing the pipeline (7 steps)
- Stage 7: Connecting the air-water cycle (3 steps)

**Figure 1.**
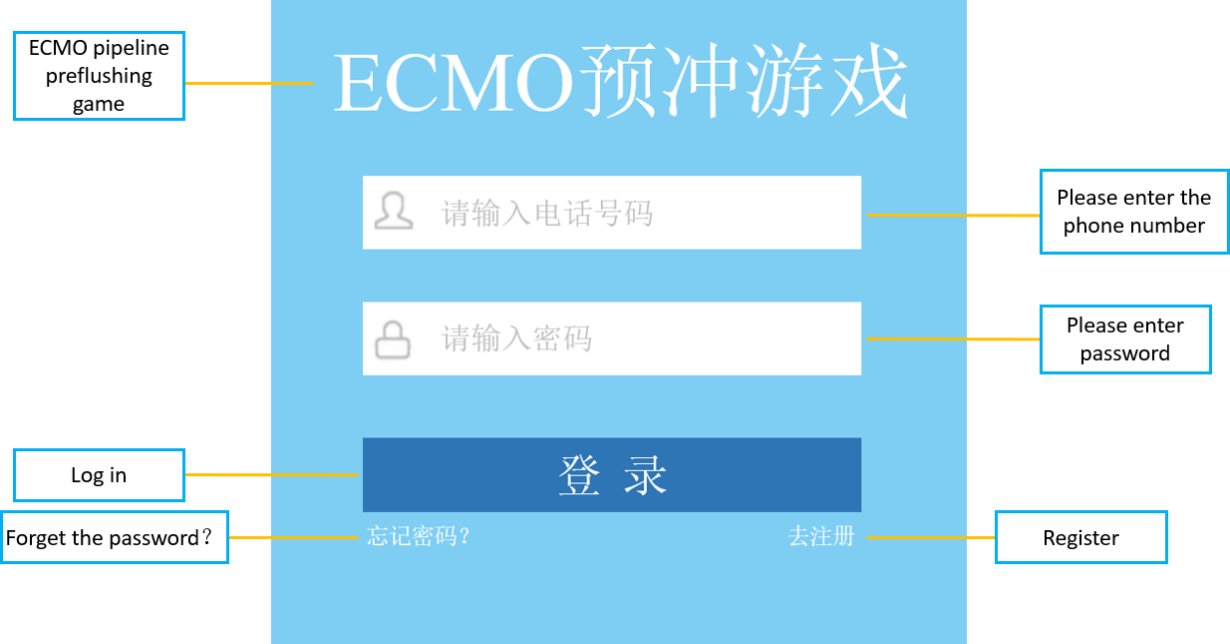
User log-in screen. ECMO: extracorporeal membrane oxygenation.

**Figure 2.**
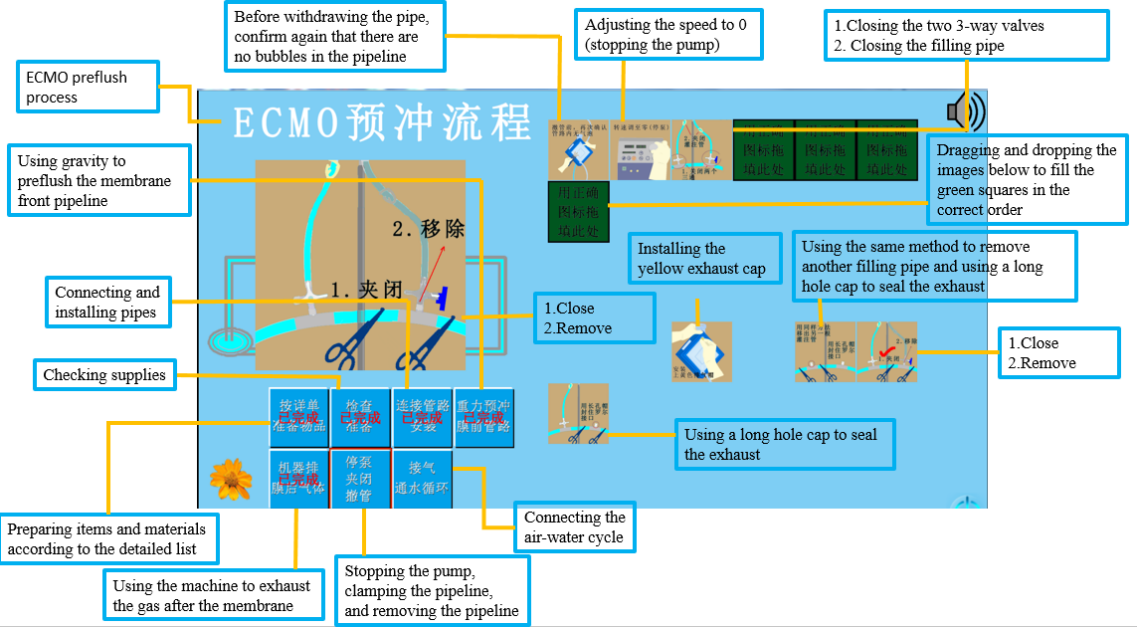
An example of the game-based mobile app. ECMO: extracorporeal membrane oxygenation.

**Figure 3.**
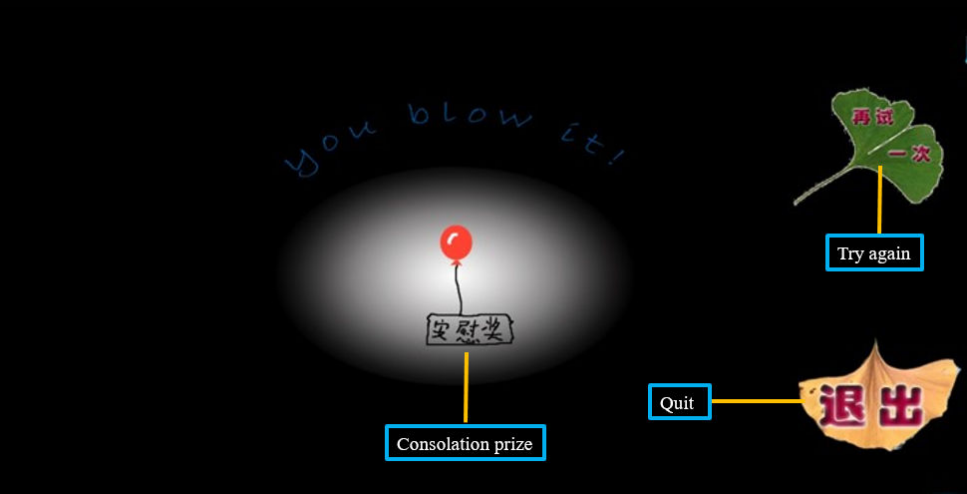
Screenshot of the interface at the end of the game when the user fails.

**Figure 4.**
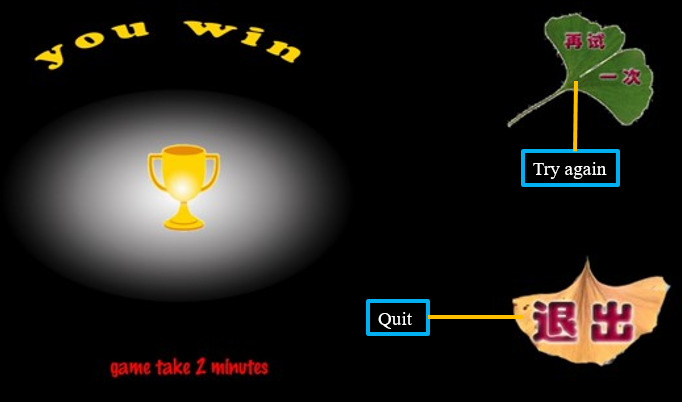
Screenshot of the interface at the end of the game when the user succeeds.

The steps for users to use the game-based mobile app are as follows. The user logs in using the registered mobile number and password; the log-in screen is shown in Figure 1. After entering the game interface, the user first needs to select the “steps” box at the bottom left of the game interface. At each step, the user need to place the cards at the bottom right of the game screen into the green boxes at the top right of the screen in the correct order. An example of the game-based mobile app is shown in Figure 2. If the user places a card in the correct position, the card remains in the box, and when the positions of all the cards and green boxes are correctly aligned, then the next step can be entered. If the card is placed in the wrong position, it will bounce back to its original position, and the flower in the lower left corner will drop a petal. The flower has 9 petals; when all the petals have dropped, the game results in failure, and the “game failure” interface is shown (Figure 3). If the user completes all the steps before all the petals fall off, the game is successful, and the “game success” interface is shown (Figure 4).

### Data Collection Tools

#### Demographic Questionnaire

A descriptive characteristics questionnaire with 5 questions was used to collect data, including sex, age, education, job title, and the number of working years.

#### Skills Evaluation Tool

The ECMO Pipeline Preflushing Theoretical Scale and ECMO Pipeline Preflushing Skills Scale were used to assess the participants’ mastery of theoretical knowledge and practical ability. We referenced the *ECMO Manual* (Chinese version; 2nd edition) [11] and *ELSO Guidelines for Training and Continuing Education of ECMO Specialists* [12] when designing these scales. The 2 scales were evaluated by 12 specialists in the field of acute and critical care and nursing education with a title of deputy senior or higher and with at least 15 years of work experience. When the content validity index–individual items (I-CVI) of a scale is >0.78 and the content validity index–overall scale (S-CVI) is >0.9, the content validity of the scale is considered good [16]. The I-CVI of the ECMO Pipeline Preflushing Theoretical Scale was 0.83, and the S-CVI was 0.91. The I-CVI of the ECMO Pipeline Preflushing Skills Scale was 0.83, and the S-CVI was 0.94.

#### ECMO Pipeline Preflushing Theoretical Scale

The ECMO Pipeline Preflushing Theoretical Scale has 58 items and consists of 3 parts: single-choice questions (40 items), multiple-choice questions (15 items), and short answers (3 items). The scale’s score ranges from 0 to 100.

#### ECMO Pipeline Preflushing Skills Scale

The ECMO Pipeline Preflushing Skills Scale has 61 items and consists of 3 parts: preparing items and materials according to the detailed list (13 item), preoperation preparation (5 items), and operation process (43 items). The scale’s score ranges from 0 to 100.

### Data Analysis Tool

Based on the results of the pre-experimental study, the researchers referred to the average number of the ECMO pipeline preflushing needed to master the skill as 25 times. Therefore, this study counted the data of 30 operations used by each participant in the experimental group. Cumulative summation analysis (CUSUM) is a sequence analysis method first proposed by Page [17] to map the learning method of operation [18]. This study used CUSUM to draw the learning curve of the experimental group using a game-based mobile app to practice ECMO pipeline preflushing operation skills. The CUSUM was calculated as follows:


$$CUSUM=\sum_{i=1}^{n}\left(X_{i}-X_{0}\right)$$

X_0_ indicates the failure rate, and X_i_ represents the procedural success or failure of each practice. If it succeeds, then X_i_=0, and if it fails, then X_i_=1. In this study, we regarded the completion of the game before all the petals fell (within 9 mistakes) as a success, and the termination of the game as a result of all the petals falling was regarded as a failure. According to previous studies [19], the operation success rate was 94.4%, so the failure rate was X_0_ = 1 – 94.4% = 5.6%.

The difference between the score of each operation of the participant and the set target value was sequentially accumulated. A learning curve was drawn, where the abscissa (*x* value) represents the number of intubation operations and the ordinate (*y* value) represents the sum of the previous cumulative sums. The curve fitting effect was evaluated by the curve fitting coefficient of determination (*R*^2^), where 0≤*R*^2^≤1. An *R*^2^ value ≥0.95 indicates that the curve fit is satisfactory. The curve derivative function formula was obtained according to the curve function formula; the curve slope value corresponding to each operation was calculated; and the corresponding abscissa (*x* value) and the curve function value when the slope *k*=0 were calculated, that is, the curve peak value. The first integer value of *x* is the minimum number of operations the research participant needed to master the skill.

### Data Analysis

The data obtained from the study were analyzed using the SPSS (version 24.0; IBM Corp) software package. The percentage, number, mean, and SD values were used in the descriptive statistical evaluation of the data. The normal or nonnormal distribution of the data was evaluated using the 1-sample Kolmogorov-Smirnov test. Nonparametric tests showed that the distribution of skill scores was nonnormal (*df*=86; *P*=.04). The skill scores between the 2 independent groups were compared using the Mann-Whitney *U* test. The *χ*^2^ test was used to analyze the relation between the grouped variants. The Wilcoxon test was used in matching groups to determine the difference in scores between the first and final operation assessments and between the first and final skill performances. The data were evaluated at a *P*<.05 significance level.

## Results

There was no significant difference in the distribution of descriptive characteristics between the control group and the experimental group (all *P*>.05; Table 1).

**Table 1.** Participant demographics between the control and experimental groups.

Demographics		Experimental group (n=43）	Control group (n=43）	*Z* score or *χ*^2^ (*df*)	*P* value
Age (y), mean (SD)		30.51 (2.520)	30.44 (3.275)	−0.327^a^	.74
Number of working years, mean (SD)		6.93 (2.414)	6.84 (2.886)	−0.435^a^	.66
**Sex, n (%)**				0.231 (1)^b^	.63
	Female	32 (74)	30 (70)		
	Male	11 (26)	13 (30)		
**Education, n (%)**				0.311 (2)^b^	.86
	Postgraduate	4 (9)	5 (12)		
	Undergraduate	36 (84)	36 (84)		
	Junior college	3 (7)	2 (4)		
**Job title, n (%)**				3.817 (2)^b^	.15
	Senior	1 (2)	2 (4)		
	Intermediate	26 (60)	17 (40)		
	Junior	16 (38)	24 (56)		

a *Z* score.

b *χ*2 value.

In the first ECMO pipeline preflushing theoretical test and skill operation test scores, no statistically significant differences were observed between the control group and the experimental group (*P*=.10 and *P*=.63). The experimental group’s final theoretical test and skill operation test scores were significantly higher than those of the control group (*P*<.001). The final theoretical test and skill operation test scores of the experimental group were significantly higher than the first test scores of the experimental group (*P*<.001). The final theoretical test and skill operation test scores of the control group were also significantly higher than the first scores of the control group (*P*<.001; Tables 2 and 3).

**Table 2.** Difference in theoretical test scores between the control and experimental groups.

Score	Experimental group, median (range)	Control group, median (range)	*Z* score	*P* value
First theoretical test scores	75.88 (66-87)	74.42 (65-90)	−1.672	.10
Final theoretical test scores	88.44 (79-95)	85.02 (78-91)	−3.972	<.001^a^
*Z* score	−5.782	−5.715		
*P* value	<.001^b^	<.001^c^		

a Difference in theoretical test scores between the control and experimental groups at the final skill performance (*P<*.05).

b Difference in theoretical test scores between the first and final skill performance in the experimental group (*P*<.05).

c Difference in theoretical test scores between the first and final skill performance in the control group (*P<*.05).

**Table 3.** Difference in skill performance scores between the control and experimental groups.

Score	Experimental group, median (range)	Control group, median (range)	*Z* score	*P* value
First skill performance scores	75.44 (67-86)	75.93 (67-88)	−0.486	.63
Final skill performance scores	89.42 (82-94)	86.33 (76-94)	−4.145	<.001^a^
*Z* score	−5.783	−5.717		
*P* value	<.001^b^	<.001^c^		

a Difference in skill test scores between the control and experimental groups at the final skill performance (*P*<.05).

b Difference in skill test scores between the first and final skill performance in the experimental group (*P*<.05).

c Difference in skill test scores between the first and final skill performance in the control group (*P*<.05).

**Figure 5.**
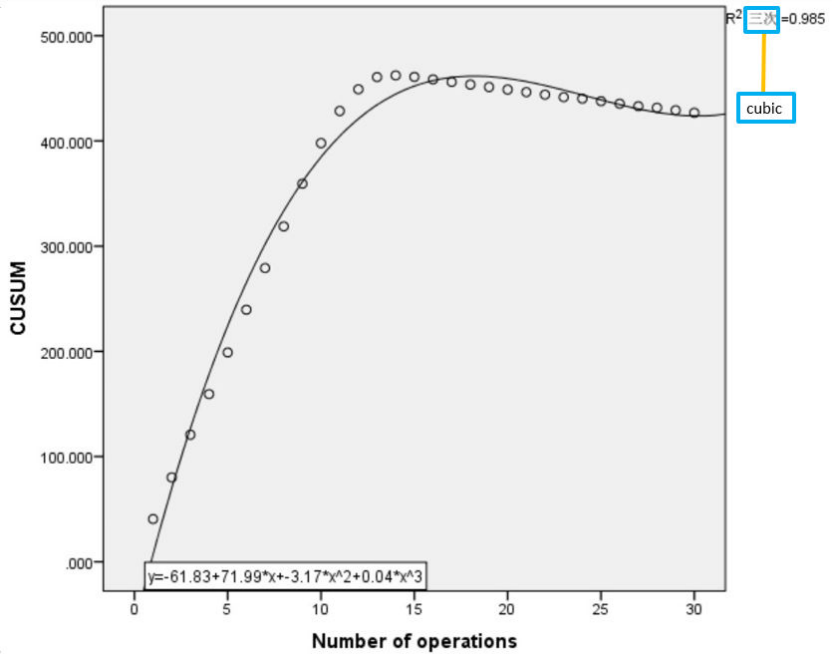
The average learning curve of the experimental group. CUSUM: cumulative summation analysis.

The fitting curve model tests of the quadratic curve, compound function curve, exponential curve, and cubic curve showed that the *P* values were all <.05, and the *R*^2^ values were 0.954, 0.498, 0.498, and 0.985, respectively. As *R*^2^>0.95 for the quadratic curve and the cubic curve, the best fitting model curve was the cubic curve (Figure 5), and the fitting equation was ∑i(CUSUM value) = 0. 04*x*^3^ – 3.17*x*^2^ + 71.99*x* – 61.83 (*x*=the number of operations). When the slope of the curve is 0, the corresponding abscissa (*x* value) represents the average number of operations for the group of students to master this skill. Calculated by the curve function formula, the first integer value of *x* after the peak of the curve of the experimental group was 17. Therefore, the minimum number of operations required for the experimental group to master this skill was 17.

## Discussion

### Principal Findings

In this study, the final averages of the ECMO pipeline preflushing theory and skill scores of the control group and the experimental group increased. The final theory and skill performance scores of the experimental group were significantly higher than those of the control group (*P*<.001). Both the game-based mobile app simulation training method and the conventional training method improved the theoretical knowledge and skill level of intensive critical care specialist nurses, but the game-based mobile app simulation training method was more effective than the conventional training method, which was consistent with the results of Gu et al [20]. In addition, the study used learning curve analysis to find that the experimental group of nurses needed 17 operations to master this skill.

Learning curves are increasingly used for health care education and formative assessment [21,22]. Every operation in the medical field requires a process from being unfamiliar to skilled. By establishing an operational learning curve, students’ mastery can be assessed [23]. It could be seen from the learning curve that in the initial stage of learning, the curve showed an upward trend and the growth rate was large. This might be because at this stage, the participants were not familiar with the operation process and the rules of the game, and the researchers set the number of mistakes to only 9 in the game. Once the number of mistakes was too high, the game was terminated. In this period, the number of successful completions of the game was relatively small, so the CUSUM value increased greatly. After the first stage of grouping and learning, as participants became familiar with the operating process, although the learning curve continued to rise, the growth rate began to decrease. We called this period the plateau period. During this period, the slope of the curve was still positive but gradually approached 0. When the learning curve began to show a downward trend, the slope of the curve began to be negative, indicating that the number of successful players in the game was gradually increasing. The reason for these results might be that after studying and gaining experience, the participants were very familiar with the operation process of ECMO pipeline preflushing and could complete the game according to the correct steps within the specified number of 9 mistakes. Therefore, the learning curve analysis showed that the nurses in the experimental group needed 17 operations to master the skill.

### Contributions and Implications

Game-based mobile apps can simulate real-life situations, and nurses or students can enter the clinical situation from the virtual world and improve practical skills and confidence before actual operations [24]. The results of this study were similar to the results of Bayram and Caliskan [25], which showed that the average score of nursing skills for pneumatic tube ostomy after using the game-based virtual reality application method was significantly improved. This app is suitable for Windows, Android, Mac, and other platforms and can be installed on computers, mobile phones, tablets, etc, thus achieving platform and network diversity. Most importantly, the game-based app provides participants with the opportunity and environment to practice and test learning outcomes anytime, anywhere, and with an unlimited number of attempts. This is different from traditional operation practices, where there is limited space, only a small number of participants can practice at a time, and the practice room is only open for a short time, along with other complicating factors. Overall, the integration of game-based mobile apps into ECMO pipeline preflushing education in China is effective in improving the learning time, knowledge, and skill level of intensive critical care specialist nurses compared to traditional training methods.

### Significance for Clinical Education

This study confirms that a game-based mobile app can help intensive critical care specialist nurses better learn and master ECMO pipeline preflushing skills. The app can help nurses translate the theoretical knowledge of nursing into practice, stimulate learners’ interest in learning, optimize the learning process, improve the learning effect, and cultivate clinical critical thinking. This app enables intensive critical care specialist nurses to learn the correct ECMO pipeline preflushing procedure for error-free operation in future clinical work while being a tool for nurses to learn and test learning outcomes anytime and anywhere. In addition, by establishing a learning curve, we understood how nurses learn a certain clinical operation for the first time and the amount of operational training required to master this operation, which has guiding significance for the development of clinical skills.

### Limitations

First, in the early stages of learning, both groups of nurses made mistakes, leading to failure, but we could not identify which steps had a high error rate. Therefore, we could not explain the reason for the high failure rate of the early operations. Second, we could not monitor the scores of the nurses in the control group during each operation practice. Therefore, we could not draw the learning curve of the control group at this time, and there was no way to compare the learning curves of the 2 groups. Third, we will use more comprehensive sampling and randomized controlled trials in future investigations. According to the follow-up feedback of the research participants, we will add related knowledge questions, related knowledge teaching videos, and scene simulation modules in the follow-up study to improve the mobile app and its attractiveness and effectiveness.

### Conclusions

This study suggests that a game-based mobile app may be more effective for intensive critical care specialist nurses in ECMO pipeline preflushing education than traditional Chinese lecture-practice education. In addition, the ECMO pipeline preflushing skill can be mastered by playing the game 17 times.

## Abbreviations

AR: augmented reality
CPR: cardiopulmonary resuscitation
CUSUM: cumulative summation analysis
ECMO: extracorporeal membrane oxygenation
I-CVI: content validity index–individual items
S-CVI: content validity index–overall scale
